# Start early! Does social instability during the pre- and early postnatal development prepare male wild cavies for social challenge later in life?

**DOI:** 10.1186/s12983-016-0187-4

**Published:** 2017-01-14

**Authors:** Katja Siegeler, Lars Lewejohann, Klaus Failing, Norbert Sachser, Sylvia Kaiser

**Affiliations:** 1 Department of Behavioural Biology, University of Muenster, Badestrasse 13, D-48149 Muenster, Germany; 2 Unit for Biomathematics and Data Processing, University of Giessen, Frankfurter Straße 95, D-35392 Giessen, Germany

**Keywords:** Adaptation, Biobehavioural profile, Cortisol, DNA fingerprinting, Prenatal, RFID, Social environment, Social experience, Testosterone, Wild cavy

## Abstract

**Background:**

The social environment the mother experiences during pregnancy and lactation can powerfully influence the offspring’s behavioural profile. Our previous studies in wild cavies show that two different social environments during pregnancy and lactation bring about different behavioural strategies of male offspring later in life: An unstable social environment leads to a behavioural camouflage strategy, hypothesised to be beneficial at times of socially challenging situations. A stable social environment during early phases of life, however, leads to an early reproduction strategy, expected to be more successful at times of social stability. In the present study, we observed the behavioural strategies of the two types of males in direct comparison in a socially challenging situation: Two adolescent males were placed simultaneously in an unknown social group consisting of one adult male and two females in a semi-naturalistic environment. Cortisol as well as testosterone concentrations and activity levels were compared. Furthermore, paternities were analysed after the males reached sexual maturity. We hypothesised that sons showing a behavioural camouflage strategy are better adapted to cope with this socially challenging situation compared to those displaying an early reproduction strategy.

**Results:**

At the beginning of the experiment, no differences in plasma cortisol concentrations between the males were found, both showed a highly significant increase due to the challenging situation. From day 5 until the end of the experiment (duration = 40 days) sons showing an early reproduction strategy had significantly higher plasma cortisol concentrations compared with those showing a behavioural camouflage strategy. Plasma testosterone concentrations did not differ significantly. Activity levels decreased significantly over time independently of the male’s behavioural strategy. Both types of males did not sire offspring during the observation period.

**Conclusion:**

Higher cortisol values from day 5 until the end of the experiment in sons showing an early reproduction strategy indicate higher levels of stress in these males compared to those displaying a camouflage strategy. We conclude that the modulation of the males behavioural strategy due to an unstable social environment during early development facilitates the endocrine adaptation to a comparable social situation later in life.

## Background

A successful adaptation to prevailing environmental conditions is crucial for the survival of an animal and its reproductive success later in life. The capacity of a specific genotype to respond to environmental cues with changes in behaviour, physiology and morphology – also called phenotypic plasticity [1] - is one important adaptive mechanism in this continuously ongoing process. Crucial life stages during ontogeny with high degrees of sensitivity towards external stimuli are of particularly importance in this non-genetic adjustment. In mammals for instance, the prenatal phase is an early sensitive phase in which modulation processes of the developing organism due to external stimuli take place (for a review, see [2]). A key factor during this life stage is the connection of the offspring to its mother via the placenta. Maternal hormones cross the placenta modifying organizational pathways of the offspring’s developing brain, which, in turn, affect physiological and behavioural responses later in life [1, 3]. This maternal control over the offspring’s development, also called maternal effects [2, 4, 5] has received much attention in evolutionary biology (see [1, 6, 7]) and is discussed to increase the variance and quality of the next generation.

The influence of the prenatal social environment on the biobehavioural profile of the offspring later in life has been investigated in a variety of species (e.g., quails: [8], mice: [9, 10], rats: [11], guinea pigs/wild cavies: [12–17], goats: [18], sows: [19], squirrel monkeys: [20, 21]. In guinea pigs, effects of unstable social environmental conditions (repeated exchange of animals between different social groups) have been thoroughly investigated: Sons whose mothers had lived in an unstable social environment during pregnancy and lactation (UE-sons) show a behavioural infantilisation compared with sons whose mothers experienced a socially stable environment during the same time (SE-sons) [13]. Further studies in wild cavies, the ancestor of the domestic guinea pig [22], could show that this phenomenon has not been brought about by artificial selection during domestication. Instead, comparable effects of social instability during pregnancy and lactation on the male wild cavy offspring’s behavioural profile can be observed: Wild cavy UE-sons exhibit more play behaviour especially at older ages compared with SE-sons, indicating an infantilised behavioural profile. This behavioural infantilisation is accompanied by a delayed increase of plasma testosterone concentrations during sexual maturity [16]. Furthermore, UE-sons display significantly lower amounts of aggressive and social orientation behaviour as well as attentive behaviour than SE-sons [16]. This slower gonadal development and juvenile behavioural profile of UE-sons has been discussed to be beneficial for young adolescent male wild cavies under socially unstable situations for the following reason [16, 17]: Male wild cavies do not tolerate other males and severely injure or kill them in agonistic encounters [23, 24]. Especially males approaching sexual maturity (i.e. around day 75 of age [25]) are often attacked by resident males [24]. In addition, amounts of social interactions and levels of aggressive behaviour are significantly heightened at times of socially unstable conditions [26–29] mainly due to the lack of stable social structures as, for instance, dominance hierarchies and social bonds between animals of the same social unit. Behaving inconspicuously by showing a juvenile behavioural profile and developing more slowly in the presence of resident adult males might decrease the risk of fights and fatal injuries for adolescent UE-sons in unstable social environmental conditions. This has been designated as a “behavioural camouflage strategy” [16, 17] and was indeed observed for adolescent male wild cavies under natural conditions in high density situations [30]. Remarkably, reproductive capacity is not altered in UE-sons compared with SE-sons [17].

In the present study, we compared the biobehavioural profiles of UE- and SE-sons in a socially challenging situation. For this purpose, we introduced one UE- and one SE-son together in an unknown semi-naturalistic environment. Within this environment, one unfamiliar resident adult male and two unknown females along with their preweaned offspring were living. This socially challenging situation with high levels of unpredictability and uncontrollability of social encounters, high amounts of social interactions and high levels of inter-male competition represents a match for the UE-sons, however, a mismatch for the SE-sons.

The following three hypotheses were investigated:

- Firstly, we hypothesised that the offspring born in these groups should be sired by a major part from the resident adult male but also for a small proportion by the better adapted sexually mature UE-sons according to moderate degrees of multiple paternities ranging from 13 to 27% known for wild cavies living under natural conditions [30].
- Secondly, we hypothesised low levels of plasma cortisol and high levels of plasma testosterone concentrations in UE-sons compared to SE-sons since different studies could show that dealing successfully with a challenging social situation is usually attended to this neuroendocrine profile [31, 32].
- Thirdly, activity levels were hypothesised to be different between the two types of males since different coping styles usually reflect different degrees of activity [33].

## Methods

### Subjects

The experiments of this study were carried out with wild cavies of the species *Cavia aperea* ERXLEBEN, 1777, derived from breeding stocks established at the Department of Behavioural Biology, University of Muenster. The animals were descendants from feral cavies trapped in the province of Buenos Aires, Argentina, in 1995 and from lineages belonging to the Universities of Bayreuth and Bielefeld, Germany. Animals of our breeding population were regularly replaced by animals stemming from different breeding stocks in Germany to ensure a reduction of potential inbreeding effects in our population. Since wild cavies do not show natural colour markings, the individuals were marked by bleaching the fur with 32% hydrogen peroxide once per month. The fur of newborn animals was bleached directly after birth for individual identification.

### Housing conditions of pregnant and lactating females

Sixteen groups were composed, consisting of one male and two female wild cavies along with their preweaning offspring. Within these groups, eight unstable as well as eight stable social environments were established (for more detail see *Establishment of unstable and stable social environments*). Each group was kept in a 1.5 m^2^ enclosure. Pups that were not used as experimental animals were removed from the enclosures at the age of 20 ± 1 days.

All animals were kept under the following standardised conditions: temperature 22 ± 2 °C, relative humidity about 55 ± 10%, light/dark cycle 12:12 h with the light phase starting at 7:00 h in the morning. Commercial guinea pig diet (Höveler “Spezialfutter” 1070 for guinea pigs, Höveler Spezialfutterwerke, Germany; altromin 3023, Altromin Spezialfutter, Germany), oat flakes (Fortin Mühlenwerke, Germany), hay and water were available *ad libitum*. Vitamin C was added to the water twice per week as it is a daily requirement in the diet of guinea pigs and wild cavies since they lack one of the enzymes that are necessary to convert glucose to ascorbic acid.

All animals were housed in wooden enclosures in our animal facility. The floors were covered with wood shavings for bedding (Allspan, Germany) and cleaned every four weeks. The enclosures were enriched with a brick, two wooden branches and two cardboards as houses for shelter.

### Establishment of unstable and stable social environments

Unstable social environment (UE): In eight groups, one of the two females was transferred to the clockwise neighbouring enclosure every second week. With a one-week shift, the other female was rotated counter-clockwise. This regularly exchange of the pregnant and lactating females between different groups led to a change in group compositions once a week. Lactating females were transferred with their offspring.

Stable social environment (SE): In contrast to unstable groups, the group composition of stable groups remained constant throughout the study. To prevent a handling bias, all females and their pups were handled in the same way at corresponding times.

The handling process of each animal of both social environmental conditions lasted approximately two minutes. It comprises catching the animal and transferring it into a new (UE) or the same (SE) social group.

### Housing conditions of the sons

The experiment was conducted with 22 sons (SE-sons: 11, UE-sons: 11) originating from the females’ second litters after the establishment of SE- and UE-groups. Second litters were used in order to ensure that the whole development of the offspring during the prenatal phase took place in established stable and unstable social environmental conditions. Since all females gave birth to offspring already before we started the manipulation of social conditions and the males were experienced breeding males, we did not expect differences in parental care between the first and second litters after the establishment of stable and unstable social conditions. All sons stayed in their mothers’ groups (stable or unstable) until day 40 ± 2 days of age. The standardised housing conditions were the same as mentioned in chapter *Housing conditions of pregnant and lactating females*.

**Fig. 1:**
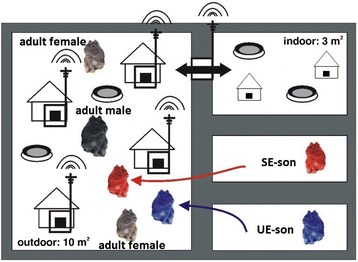
Graphical representation of the semi-naturalistic environment consisting of a less sheltered outdoor part and a more sheltered indoor part. The figure shows the unfamiliar social group where UE- and SE-sons were placed in (UE-sons: sons whose mothers had lived in an unstable social environment during pregnancy and lactation; SE-sons: sons whose mothers had lived in a stable social environment during pregnancy and lactation). Blue cavy: SE-son, red cavy: UE-son. Big cavy in the middle of the less sheltered outdoor part: adult male, cavies at the top and the bottom: adult females. RFID-antennae for measuring activity were located around plastic tubes in front of each of the four wooden houses of the less sheltered outdoor part and around a plastic tube that connected the two parts of the semi-naturalistic environment via a ramp

At day 40 ± 2 days of age, one SE- and one UE-son were placed simultaneously into an unknown enclosure (Fig. 1). In each enclosure, an unfamiliar social group consisting of one adult male and two adult females along with their preweaned offspring were already living at least for one week before the introduction of the two males took place. The offspring was removed from the enclosures at the age of 20 ± 1 days.

All enclosures were located outside in order to simulate semi-natural conditions. Temperature, relative humidity and the light/dark cycle followed prevailing natural conditions including spring, summer, autumn and winter conditions. Each enclosure was about 13 m^2^ and consisted of a less sheltered outdoor part and a more sheltered indoor part (less sheltered outdoor part 10 m^2^, more sheltered indoor part 3 m^2^). They were connected via a ramp and a plastic tube and enriched with hiding places (2 cardboards and 4 wooden houses) and wooden branches. In addition, the less sheltered outdoor part was subdivided into different areas by plastic walls. This additional fragmentation was important for a successful introduction of SE- and UE-sons in this new social groups since it is known for wild cavies that adult males and females can be highly aggressive against non-related animals [23, 24]. During wintertime, a heat radiator was placed into the more sheltered indoor parts of each enclosure to ensure that the animals had the chance to warm-up in a heated area.

All animals were fed in the same way as described above (see *Housing conditions of pregnant and lactating females*). Floors of the more sheltered indoor parts were covered with wood shavings, floors of the less sheltered outdoor parts were covered with sand. The whole enclosure was cleaned every four weeks.

### Measurement of activity of the animals via radio-frequency identification

The radio-frequency identification (RFID) method used in this study allowed a computer-based analysis of activity of the animals of each social group of the outdoor enclosures. For this purpose, all animals carried an RFID-transponder (Type ID 100, TROVAN, Ltd.) under the skin of their neck. The transponder were injected subcutaneously under isoflurane anesthesia by using a specially designed injector (IID-100). The injection took place for UE- and SE-sons after weaning. In addition, two coiled antennae were placed around a plastic tube in front of each of the four wooden houses of the less sheltered outdoor parts as well as at the ramp which connected the less sheltered outdoor and the more sheltered indoor part. By passing the antennae, an electromagnetic field was induced which, in turn, leads to a transmission of the ID of the passing animal towards two reading devices (Typ LIDD665N, TROVAN, Ltd.). IDs of passing animals and time stamps were automatically registered by a SQL database over the whole observation period. For analyses, the observation period (in total 60 days) was summarised in intervals of 10 days: day 1–10, day 11–20, day 21–30, day 31–40, day 41–50 and day 51–60.

### Blood sampling and body weights

Blood samples were collected at day 40 ± 2 of age directly before and 2 h after the introduction of SE- and UE-sons into the new social groups. The latter blood sample was collected in order to investigate the short-term reaction of the animals to this social challenge since it is known that maximum values of cortisol in wild cavies are reached on average two hours after the onset of a stressor [34]. The long-term influence on cortisol and testosterone concentrations due to this social challenge was investigated by sampling blood on day 5, 20, 30 and 40 after the introduction had taken place.

All blood samples were taken at a fixed point in time set at 13.00 h ± 15 min in order to avoid possible influences of circadian rhythm in hormone concentrations (domestic guinea pigs show diurnal variations in plasma cortisol titres with a peak around 13:00 h [35]). An increase in cortisol levels at this time point can be assigned to the challenging situation and is not related to circadian influences anymore since maximum values are already reached. The blood used for cortisol determination was collected within the first three minutes, blood samples for testosterone analysis within six minutes after entering the enclosure. Both, cortisol and testosterone concentrations are known to be unaffected by the occurrence of stress during this short time interval [35]. Blood samples were collected from the blood vessels of the ears. During blood sampling, one experimenter held the animal in his/her lap, while a second person collected the sample. In a first step, a heat rub (Finalgon, Boehringer Ingelheim Pharma, Germany) was applied to the ear of the animal to stimulate the blood circulation. After removing the salve, the vessels were illuminated with a hand-held LED light and pricked with a sterile injection needle. About 0.02 - 0.05 ml blood was collected in heparinized capillary tubes (Brand, Germany). Afterwards, the plasma was separated by centrifugation (11.700 ×*g* for 7 min) and subsequently deep-frozen at −20 °C until assayed. After each blood sampling, the body weights were measured.

### Endocrine analyses

Plasma cortisol concentrations were analysed in duplicate using a luminescence immunoassay (Cortisol luminescence immunoassay RE62011, IBL International, Germany). The antibodies used cross-reacted with relevant steroids as follows: cortisol 100%, prednisolone 30%, 11-deoxycortisol 8.5%, cortisone 4.5%, prednisone 2.1%, corticosterone 2.0%, and 6ß-hydroxycortisol 1.0%. The intra- and interassay coefficients of variation were 4% and 7.2%, respectively.

Plasma testosterone concentrations were determined in duplicate using a solid phase enzyme-linked immunosorbent assay (ELISA; Testosterone ELISA Kit, Demeditec Diagnostics, Germany). The antibody cross-reacted with relevant steroids as follows: testosterone 100%, 11β-hydroxytestosterone 3.3%, 19-nortestosterone 3.3%, androstenedione 0.9%, 5α-dihydrotestosterone 0.8%, 17α-methyltestosterone 0.1%, epitestosterone, oestradiol, progesterone, cortisol, oestrone and danazol < 0.1% each. The intra- and interassay coefficients of variation were 5.74% and 7.23%, respectively.

### Paternity tests

SE- as well as UE-sons stayed in the new social groups until both females of each group gave birth to their offspring after SE- and UE-sons had reached sexual maturity (i.e. around day 75 of age [25]). Since female wild cavies show a postpartum oestrus [36], all three males of one group (one adult male, one SE- and one UE-son) had the chance to mate once with each female. Paternities of in total 51 pups were determined by directly comparing alleles between potential fathers and offspring born in the groups as the genotypes of the potential fathers and the mothers were known. For analyses, tissue samples of the ear were collected via an ear punch after weaning. Genomic DNA was purified by first digesting the tissue samples using protein kinase, followed by phenol/chloroform extraction and DNA precipitation with ethanol. DNA pellets were washed with 70% ethanol several times and re-suspended in TE-buffer. Eleven microsatellites were amplified by PCR [30, 37] and sequenced. Alleles were analysed using GeneMarker®.

### Statistical analysis

The data of plasma cortisol and testosterone concentrations as well as body weights were analysed using a generalized linear mixed model analysis with hierarchical random effects (GLMM, package “lme4”, routine “lmer”, R version 3.1.1 (2014-07-10)) where treatment (stable or unstable), experimental day and litter size (covariate) were fixed factors and pair ID a random factor. The model was fitted to the data by maximum likelihood techniques and statistically tested for significance by Wald test. The statistical method was used for cortisol and testosterone since this kind of analysis is able to deal with rare missing values at different time points, which occurred within this study due to technical reasons during our blood sampling procedure and the following analyses. No significant influence of pair ID could be revealed.

Activity data were analysed using a two-way analysis of covariance with repeated measures based on the effects of treatment and time (repeated measures factor) and the covariate litter size (Statistical program package BMDP, [38]). A log transformation of activity data and cortisol and testosterone data was performed since their statistical distributions were skewed to the right and deviated from a normal distribution shape. This was inspected by residual analyses by means of Q-Q-plots and histograms (R routine “3.1.1 (2014-07-10)”). Three animals per group (three UE- and three SE-sons) had to be excluded from the analysis due to missing values.

SigmaPlot 10.0 for Windows software (SPSS Inc., Chicago, USA) was used to create graphs. These show the means and standard errors of activity data and plasma cortisol and testosterone concentrations. In general, for each observed parameter in each phase of the experiment, a statistical significance level of α = 0.05 was used to test the two-sided hypotheses (first kind error rate related to each comparison).

## Results

### Activity

**Fig. 2:**
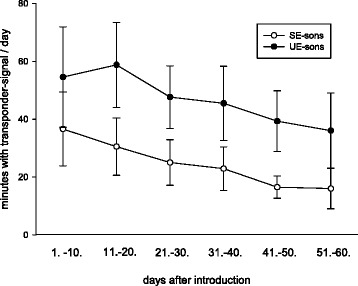
Activity (minutes with transponder signal) on day 1.-10., 11.-20., 21.-30., 31.-40., 41.-50. and 51.-60. after introduction of SE- and UE-sons into the unfamiliar social groups. SE-sons: sons whose mothers had lived in a stable social environment during pregnancy and lactation; UE-sons: sons whose mothers had lived in an unstable social environment during pregnancy and lactation. Data are shown as means and standard error. Decrease of activity of SE- and UE-sons over time from day 1 to day 60: Two-way analysis of covariance with repeated measures, *p* = 0.008. Comparison of activity between the two groups from day 1 to day 60: Two-way analysis of covariance with repeated measures, *p* = 0.117. All statistics were two-tailed, n_SE-sons_ = 8, n_UE-sons_ = 8

Overall, there was a large variation between individual wild cavies regarding the total number of antenna contacts. Therefore, for each minute it was evaluated whether or not an antenna contact occurred. Minutes with at least one antenna contact were counted as active minutes (= active animal) while minutes with no antenna contact were considered as inactive minutes (inactive animal). The statistical analyses revealed that activity of SE- and UE-sons decreased significantly over time (Fig. 2, factor time: *p* = 0.008, *F* = 3.80, df = (5;35)). However, activity levels did not differ significantly between the two types of males (Fig. 2, factor treatment: *p* = 0.117, *F* = 3.34, df = (1;6)). Furthermore, there was no statistically significant interaction between the effects of the early rearing conditions (stable or unstable) and the time course of the experiment (Fig. 2, factor interaction: *p* = 0.551, *F* = 0.81, df = (5;35)).

### Endocrine parameters

**Fig. 3:**
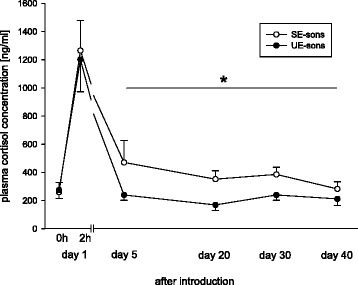
Plasma cortisol concentrations [ng/ml] directly before (basal value) and two hours after the introduction of SE- and UE-sons into the unfamiliar social groups (reaction value) and subsequently following on day 5, day 20, day 30 and day 40. SE-sons: Sons whose mothers had lived in a stable social environment during pregnancy and lactation; UE-sons: Sons whose mothers had lived in an unstable social environment during pregnancy and lactation. Data are shown as means and standard error. Increase after two hours after the introduction of SE- and UE-sons in the unfamiliar social groups: GLMM, *p* = 0.001. Comparison of this increase between the two groups GLMM, *p* = 0.915. Comparison of the two groups on day 5, day 20, day 30 and day 40: GLMM, *p* = 0.043. All statistics were two-tailed, n_SE-sons_ = 8–11, n_UE-sons_ = 9–11

At the onset of the experiment, plasma cortisol concentrations of SE- and UE-sons did not differ (Fig. 3, factor treatment: GLMM/Wald test, *p* = 0.881). Two hours after introduction into the unfamiliar social groups, plasma cortisol concentrations in SE- and UE-sons rose significantly by a factor of more than 5 (Fig. 3, GLMM: *t*-value = 5.72, *p* < 0.001) due to the unknown social and environmental situation. This increase did not differ significantly between the two groups (Fig. 3, GLMM: *t*-value = −0.11, *p* = 0.915). Contrary, from day 5 until the end of the experiment, SE-sons showed significantly higher plasma cortisol concentrations compared with UE-sons (Fig. 3, factor treatment: GLMM/Wald test, *p* = 0.043). No significant effect regarding time (Fig. 3, factor time: GLMM/Wald test, *p* = 0.483) nor interaction between time and treatment could be revealed (Fig. 3, factor interaction: GLMM/Wald test, *p* = 0.570).

**Fig. 4:**
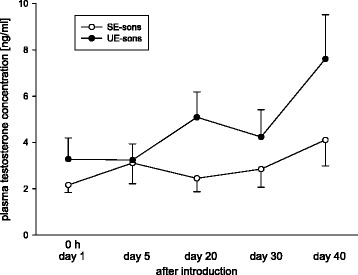
Plasma testosterone concentrations [ng/ml] on day 5, day 20, day 30 and day 40 after introduction of SE- and UE-sons into the unfamiliar social groups. SE-sons: sons whose mothers had lived in a stable social environment during pregnancy and lactation; UE-sons: sons whose mothers had lived in an unstable social environment during pregnancy and lactation. Data are shown as means and standard error. Comparison of the two groups on day 5, day 20, day 30 and day 40: GLMM, *p* = 0.912. Statistics were two-tailed, n_SE-sons_ = 6–8, n_UE-sons_ = 8–11

Regarding testosterone, plasma concentrations did not differ significantly between SE- and UE-sons at the onset of the experiment (Fig. 4, factor treatment: GLMM/Wald test, *p* = 0.245). Furthermore, no significant differences between the two groups could be revealed from day 5 to day 40 after introduction in the unknown social groups (Fig. 4, factor treatment: GLMM/Wald test, *p* = 0.912). In addition, time (Fig. 4, factor time: GLMM/Wald test, *p* = 0.854) and the interaction between treatment and the time course of the experiment did not differ significantly (Fig. 4, factor interaction: GLMM/Wald test, *p* = 0.343).

### Body weights

Body weights did not differ significantly between SE- and UE-sons at the onset of the experiment (factor treatment: GLMM/Wald test, *p* = 0.918; data not shown). After their introduction in the unknown social groups, UE- as well as SE-sons showed a highly significant increase in body mass from day 1 until day 40 (factor time: GLMM/Wald test, *p* < 0.001; data not shown). This increase in body mass did not differ significantly between the two types of males over the whole observation period (factor treatment: GLMM/Wald test, *p* = 0.617; data not shown). Furthermore, the interaction between treatment and the time course of the experiment did not differ significantly (factor interaction: GLMM/Wald test, *p* = 0.701; data not shown).

### Paternity tests

In total, paternities of 51 pups were analysed. By comparing genotypes of these pups with all potential fathers of the present study (adult male, SE- or UE-son) it could be revealed that all offspring were sired exclusively by the resident adult male. Neither UE- nor SE-sons sired offspring during the whole observation period.

## Discussion

In the present study, two different types of male wild cavies raised under two different social environmental conditions during their pre- and early postnatal development (stable or unstable) were confronted within a highly socially challenging situation. At early adolescence, they were placed together in an unfamiliar social group consisting of one adult male and two adult females along with their preweaned offspring living in a semi-naturalistic environment. The social situation within this semi-naturalistic environment represented a match for the UE-sons (high levels of unpredictability and uncontrollability of social encounters, high amounts of social interactions and high levels of inter-male competition), however, a mismatch for the SE-sons (for more details see introduction). Our results show that UE-sons showed significantly lower plasma cortisol concentrations in this highly socially challenging situation compared to SE-sons. Testosterone concentrations, body weights, activity levels and paternities did not differ significantly between the two types of males.

### Neuroendocrine profiles, coping styles and adaptation

In challenging situations, e.g. attacks by a predator or competition between individuals, two main stress axes, the sympathetic-adrenomedullary (SAM) and the hypothalamic-pituitary-adrenocortical (HPA) axis are immediately activated [39]. An activation of the SAM axis leads to an increase in catecholamines as adrenalin and noradrenalin, which, in turn, increase, for instance, heart rate, blood pressure and breathing rate of an animal. An activation of the HPA axis leads to an increased excretion rate of glucocorticoids as cortisol and corticosterone, which provides the animal with energy by increasing gluconeogenesis and lipolysis [32]. A long-term activation of the SAM and HPA axis, however, is related to serious diseases such as diabetes, hypertension, arteriosclerosis, osteoporosis, psychosis, impaired immune function or depression [32].

In their natural habitat, wild cavies are frequently exposed to challenging situations, which lead to an activation of the SAM and the HPA axis. They range from seasonal changes in climatic factors such as temperature, precipitation to high levels of predation pressure or intraspecific competition [30, 40, 41]. In our study, we mimicked a very common challenging environment under natural conditions, i.e. social instability and high levels of inter-male competition. This situation is particularly challenging for young adolescent males since adult male wild cavies usually behave very aggressively against other especially younger ones [23, 24].

As expected, the HPA-axis was activated in UE- as well as in SE-sons, i.e. a strong increase of plasma cortisol concentrations occurs within two hours after being introduced into the unfamiliar social groups. During this acute challenging situation, values did not differ significantly between the two types of males. However, already within five days after the introduction of UE- and SE-sons, cortisol levels of UE-sons had reached basal levels again indicating that these males already interacted peacefully and in a non-stressful way with this new social environmental situation contrarily to SE-sons. The latter showed significantly higher plasma cortisol values over the whole experimental phase and reached basal values for the first time at the end of the observation period (i.e. around 40 days after their introduction into the unfamiliar social groups). Our data does not allow to draw conclusions whether or not a negative effect of higher cortisol levels in SE-sons occurred. The results show, however, that SE-sons needed significantly longer to cope efficiently with this unknown challenging social situation compared to UE-sons. In contrast, UE-sons adapted more rapidly to this social situation of unpredictability and uncontrollability of social encounters and high levels of inter-male competition. This confirms our hypothesis that showing a behavioural camouflage strategy is indeed promising for young adolescent males in the presence of resident adult males.

Moreover, we hypothesised also a significant influence on plasma testosterone concentrations in both types of males since the neuroendocrine profile of socially challenged animals comprises not only effects on plasma cortisol, but also on testosterone concentrations [42–44]. More specifically, we expected higher levels of plasma testosterone concentrations in UE-sons compared to SE-sons. Former studies show that the developmental time course of testosterone differs significantly between the two types of males with UE-sons showing a slower increase in testosterone concentrations around sexual maturity compared to SE-sons [16, 17]. The results of the present study show that plasma testosterone concentrations did not differ significantly between the two types of males. It might be, however, that significant differences exist at older ages.

Furthermore, we initially hypothesised that different coping strategies of differentially raised males would be reflected in different activity profiles. Unfortunately, our method of measuring activity by a limited number of antennae was not sensitive enough to reveal any significant differences between the male types. The number of antenna contacts, however, decreased significantly in both types of males during the course of the study indicating an overall reduction in daily activity possibly due to an adaptation to the unknown environmental conditions and an integration into the previously unknown social groups. In addition, the seasonal variation could have increased the variability. This might be another reason why possible significant differences in testosterone and activity between SE- and UE-sons could have remained undetected in our study.

### Paternities

Paternities of in total 51 pups born in the experimental groups were determined by directly comparing alleles between potential fathers (adult male, UE- or SE-sons) and offspring born in these groups. The results of the present study show that exclusively the resident adult males of each group sired offspring successfully. This is somehow surprising since a field study of the wild cavies ecology and social mating system revealed under natural conditions moderate degrees of multiple paternities ranging from 13 to 27% [30]. However, it is also known from this study that only large males with more than 500 g occupy stable home ranges successfully with access to females. An explanation for the lack of paternities for UE- and SE-sons in the present study might be that the restricted housing conditions of our semi-naturalistic environment enabled the resident males to defend their females always successfully against the younger and smaller males, which were still in growth and weighed less than 500 g until the end of the experiment.

## Conclusion

Taken together, young adolescent UE-sons seem to be better adapted to cope with a socially challenging environmental situation than SE-sons although we have to admit that this conclusion is based on differences in cortisol concentrations only. We hypothesise that these effects are primarily brought about by female wild cavies adapting their offspring to the social situation they experienced during pregnancy and lactation. From an evolutionary point of view, it seems likely that these maternal effects [2, 4, 5] have evolved through natural selection.
